# mTORC1 Crosstalk With Stress Granules in Aging and Age-Related Diseases

**DOI:** 10.3389/fragi.2021.761333

**Published:** 2021-10-13

**Authors:** Marti Cadena Sandoval, Alexander Martin Heberle, Ulrike Rehbein, Cecilia Barile, José Miguel Ramos Pittol, Kathrin Thedieck

**Affiliations:** ^1^ Institute of Biochemistry and Center for Molecular Biosciences Innsbruck, University of Innsbruck, Innsbruck, Austria; ^2^ Laboratory of Pediatrics, Section Systems Medicine of Metabolism and Signaling, University of Groningen, University Medical Center Groningen, Groningen, Netherlands; ^3^ Department for Neuroscience, School of Medicine and Health Sciences, Carl von Ossietzky University Oldenburg, Oldenburg, Germany

**Keywords:** MTOR, aging hallmarks, stress, insulin, amino acids, cellular signaling, stress granules (SGs), autophagy

## Abstract

The mechanistic target of rapamycin complex 1 (mTORC1) kinase is a master regulator of metabolism and aging. A complex signaling network converges on mTORC1 and integrates growth factor, nutrient and stress signals. Aging is a dynamic process characterized by declining cellular survival, renewal, and fertility. Stressors elicited by aging hallmarks such as mitochondrial malfunction, loss of proteostasis, genomic instability and telomere shortening impinge on mTORC1 thereby contributing to age-related processes. Stress granules (SGs) constitute a cytoplasmic non-membranous compartment formed by RNA-protein aggregates, which control RNA metabolism, signaling, and survival under stress. Increasing evidence reveals complex crosstalk between the mTORC1 network and SGs. In this review, we cover stressors elicited by aging hallmarks that impinge on mTORC1 and SGs. We discuss their interplay, and we highlight possible links in the context of aging and age-related diseases.

## Introduction

The mechanistic target of rapamycin (MTOR) is a serine/threonine protein kinase conserved across all eukaryotes (Tatebe and Shiozaki, 2017). MTOR constitutes a central hub that integrates metabolic signals and adapts cellular processes to extrinsic and intrinsic changes and stressors in health, disease, and aging (Papadopoli et al., 2019; Liu and Sabatini, 2020).

MTOR resides in two complexes, MTOR complex 1 and 2 (mTORC1 and mTORC2), each of which regulates distinct functions in metabolic control throughout the life course [reviewed by Liu and Sabatini (2020) and Papadopoli et al. (2019)]. The two complexes exhibit different sensitivities to the macrolide rapamycin that gave MTOR its name (Heitman et al., 1991; Brown et al., 1994; Sabatini et al., 1994). Rapamycin directly binds and inhibits mTORC1 (Yang et al., 2013; Aylett et al., 2016) whereas long-term rapamycin exposure indirectly inhibits also mTORC2 (Sarbassov et al., 2006). From yeast to mammals, rapamycin extends lifespan, highlighting the fundamental role of MTOR as a regulator of longevity and aging (Weichhart, 2018; Papadopoli et al., 2019). Aging is a dynamic process whereby physiological functions needed for survival, renewal, and fertility deteriorate over time. Its pace differs among species and individuals due to differences in molecular networks and in stochastic damage of cellular components (Khan et al., 2017). Mitochondrial malfunction, loss of proteostasis, genomic instability and telomere shortening, dysregulated nutrient sensing, and altered cell communication are considered as hallmarks of aging (Lopez-Otin et al., 2013) and are directly regulated by MTOR [reviewed in detail by Papadopoli et al. (2019)]. Conversely, MTOR also responds to their dysregulation, thus contributing to age-related processes upstream and downstream of aging hallmarks. In this review, we cover stressors elicited by aging hallmarks that impinge on the signaling network converging on mTORC1. We highlight the complex crosstalk of mTORC1 with the formation of stress granules (SGs), a stress-dependent non-membranous cellular compartment, and we discuss the impact of their interplay in aging and age-related diseases.

## The mTORC1 Network Under Nutrient Sufficiency and Stress

### mTORC1 Activation by Growth Factors

**FIGURE 1:**
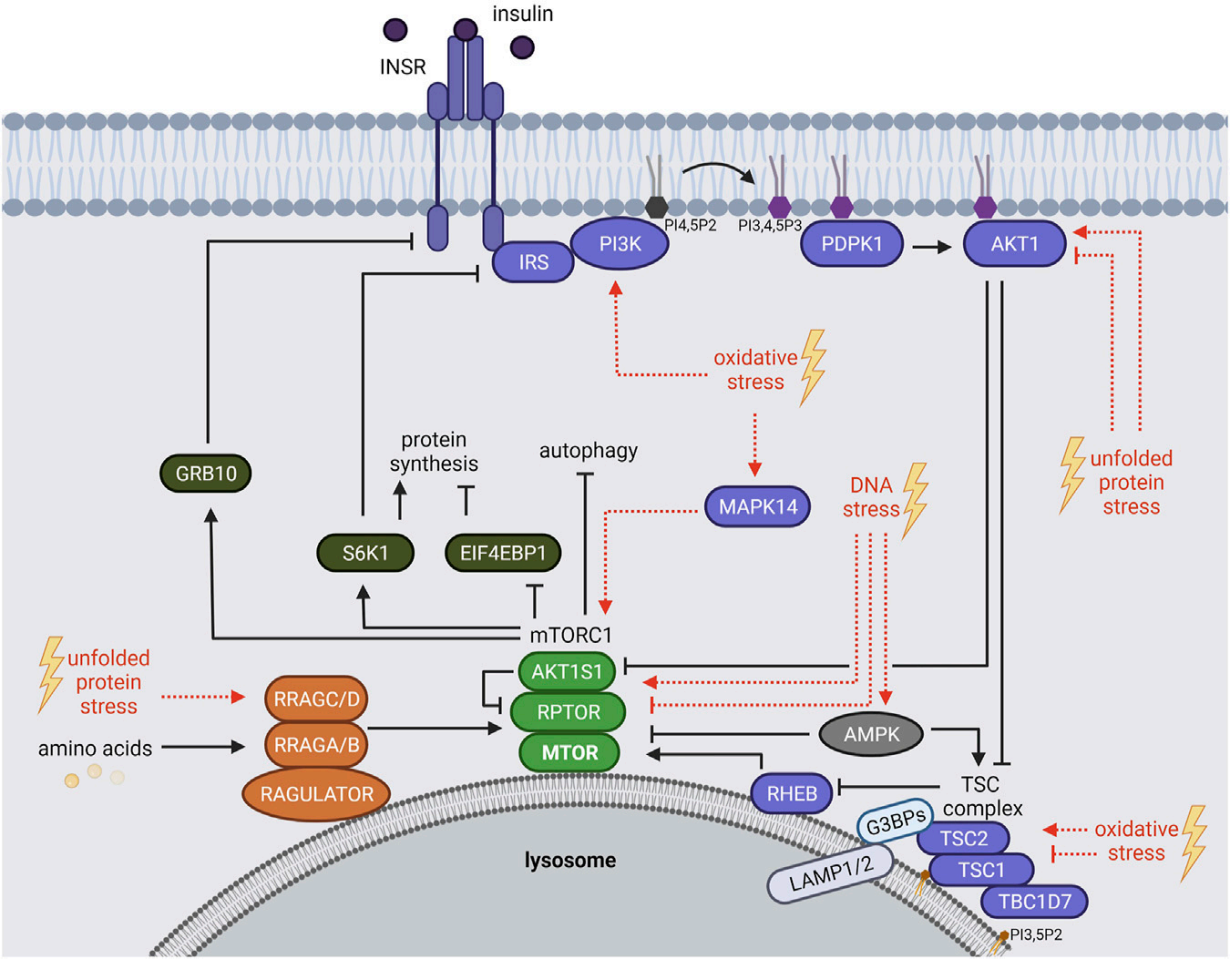
The mTORC1 signaling network. mTORC1 is regulated by growth factors (insulin), amino acids and stressors. Insulin binds and activates the INSR, which recruits IRS and PI3Ks. PI3Ks convert PI4,5P2 to PI3,4,5P3 that serves as an anchor for PDPK1 and AKT1 at the plasma membrane. PDPK1 activates AKT1, which in turn inhibits AKT1S1 and the TSC complex. Hence, TSC-mediated RHEB inhibition is repressed resulting in mTORC1 activation at the lysosome. Amino acids regulate mTORC1 recruitment to the lysosomal surface mainly through the RRAG GTPases. mTORC1 phosphorylates many substrates including EIF4BP1, RPS6KB1 (S6K1) and GRB10. S6K1 and GRB10 mediate negative feedback loops towards the IRS and the INSR, respectively. Age-related stressors (depicted in red) activate or inhibit the mTORC1 network via different mechanisms. See main text for details and abbreviations.

mTORC1 responds to a plethora of environmental cues, including growth factors [e.g. insulin or insulin like growth factor 1 (IGF-1)], nutrients (e.g. amino acids) and stressors (Figure 1) (Heberle et al., 2015; Ben-Sahra and Manning, 2017; Gonzalez and Hall, 2017; Liu and Sabatini, 2020). Upon insulin or IGF-1 binding, the insulin receptor (INSR) or insulin like growth factor 1 receptor (IGF1R) auto-phosphorylate their cytoplasmic domains (Vigneri et al., 2016; Razquin Navas and Thedieck, 2017), allowing the recruitment of several insulin receptor substrate (IRS) protein isoforms (Sun et al., 1991; Razquin Navas and Thedieck, 2017). The INSR phosphorylates the IRS at tyrosine residues, which in turn act as scaffolds for other proteins, including phosphatidylinositol 3-kinases (PIK3C, also known as PI3Ks) (Hadari et al., 1992). Class I PI3Ks convert phosphatidylinositol-4,5-bisphosphate (PI4,5P2) to phosphatidylinositol-3,4,5-trisphosphate (PI3,4,5P3), serving as an anchor site for proteins at the plasma membrane (Czech, 2000; Dibble and Cantley, 2015; Bilanges et al., 2019; Hoxhaj and Manning, 2020) (Figure 1). The pleckstrin homology (PH) domains of PDPK1 (phosphoinositide-dependent kinase-1) and AKT1 (AKT serine/threonine kinase 1) both bind to PI3,4,5P3 (Anderson et al., 1998; Vanhaesebroeck et al., 2012; Dibble and Cantley, 2015). PDPK1 phosphorylates and activates AKT1 (Alessi et al., 1996; Alessi et al., 1997). In turn, AKT1 inhibits the mTORC1 suppressors AKT1 substrate 1 (AKT1S1, also known as PRAS40) (Kovacina et al., 2003; Haar et al., 2007; Nascimento et al., 2010) and the tuberous sclerosis (TSC) protein complex (Inoki et al., 2002). The TSC complex contains the TSC complex subunit 1 (TSC1, also known as hamartin), TSC2 (also known as tuberin), and TBC1 domain family member 7 (TBC1D7) (Dibble et al., 2012; Ramlaul et al., 2021; Yang et al., 2021). When AKT1 is inactive, the TSC complex inhibits mTORC1 at the lysosomes, constituting its central signaling platform (Carroll and Dunlop, 2017). TSC2 harbors a GTPase activating protein (GAP) function towards the small GTPase RHEB (Ras homolog, mTORC1 binding) (Inoki et al., 2003; Tee et al., 2003b; Zhang et al., 2003) (Figure 1). When AKT1 is activated by insulin, it phosphorylates TSC2 (Inoki et al., 2002; Menon et al., 2014), and the lysosomal localization of the TSC complex is reduced (Menon et al., 2014). Also other growth factor responsive pathways including MAPK (mitogen-activated protein kinase) (Tee et al., 2003a; Ma et al., 2005) and WNT (Wnt family member) (Inoki et al., 2006) converge on TSC2, leading to its phosphorylation and inactivation. GTP-bound RHEB binds and activates mTORC1 at the lysosomal surface (Castro et al., 2003; Garami et al., 2003; Inoki et al., 2003; Tee et al., 2003b; Zhang et al., 2003; Long et al., 2005). mTORC1 restricts its own activity via several negative feedback loops (Figure 1) mediated by the mTORC1 substrates RPS6KB1 (ribosomal protein S6 kinase B1, also known as S6K1) that phosphorylates and inhibits the IRS (Um et al., 2004; Tzatsos and Kandror, 2006), and GRB10 (growth factor receptor-bound protein 10) causing the inhibition of the INSR (Hsu et al., 2011; Yu et al., 2011).

### mTORC1 Response to Amino Acids

Amino acids mediate the lysosomal recruitment of mTORC1 (Kim et al., 2008; Sancak et al., 2008; Sancak et al., 2010). This finding explains the long-known hierarchy of insulin and amino acid signals to mTORC1 whereby the insulin-RHEB axis can only activate mTORC1 in the presence of amino acids (Hara et al., 1998), when mTORC1 resides at the lysosomes (Sancak et al., 2010; Carroll and Dunlop, 2017). The machineries recruiting mTORC1 to lysosomes are complex and converge mainly on the four Ras related GTP binding GTPases (RRAG A, B, C, D) (Figure 1) [reviewed by Gonzalez and Hall (2017), Wolfson and Sabatini (2017), Kim and Guan (2019), Carroll (2020)]. In their active form, GTP-bound RRAGA or RRAGB and GDP-bound RRAGC or RRAGD assemble as heterodimers (A or B with C or D). They bridge mTORC1 to the RAGULATOR complex [LAMTOR, formed by the late endosomal/lysosomal adaptor MAPK and MTOR activator proteins 1-5, (LAMTOR 1-5)] which resides at the lysosomal surface (Teis et al., 2002; Sancak et al., 2010; Bar-Peled et al., 2012; de Araujo et al., 2017). Specific sensors transduce distinct amino acid signals that activate the RRAGs (Figure 1). For example, 1) leucine is sensed by SESN2 (sestrin 2) (Chantranupong et al., 2014; Parmigiani et al., 2014; Peng et al., 2014; Saxton et al., 2016b; Wolfson et al., 2016) and enhances acetylation of the mTORC1 scaffold protein RPTOR (regulatory associated protein of MTOR complex 1, also known as raptor) and mTORC1-RRAGs binding (Son et al., 2019). 2) Arginine is sensed by CASTOR1 (cytosolic arginine sensor for mTORC1 subunit 1) (Chantranupong et al., 2014; Saxton et al., 2016a) and SLC38A9 (solute carrier family 38 member 9) (Jung et al., 2015; Rebsamen et al., 2015; Wang et al., 2015), 3) S-adenosylmethionine (SAM) by BMT2 (base methyltransferase of 25S rRNA 2 homolog, also known as SAMTOR) (Gu et al., 2017; Kitada et al., 2020), and 4) glutamine and leucine activate mTORC1 via α-ketoglutarate, produced by glutaminolysis, in a RRAG-dependent manner (Duran et al., 2012). Amino acids also activate mTORC1 via RRAG-independent routes (Melick and Jewell, 2020). For instance, glutamine and asparagine signal to lysosomal ATP6V0D1 (ATPase H+ transporting V0 subunit d1) and ARF1 (ADP ribosylation factor 1), thereby promoting mTORC1 activation through a yet unknown mechanism (Stracka et al., 2014; Jewell et al., 2015; Bernfeld et al., 2018; Melick and Jewell, 2020; Meng et al., 2020; Takahara et al., 2020).

Not only mTORC1 but also the TSC protein complex shuttles between the cytoplasm and the lysosomal surface. Recent evidence has shed light on the underlying molecular mechanisms: 1) TSC2 is tethered by the G3BP stress granule assembly factors 1 and 2 (G3BP1 and G3BP2, G3BPs) to the cytoplasmic portion of the lysosomal associated proteins 1 and 2 (LAMP1/2) (Prentzell et al., 2021) (Figure 1). Also the RHEB and RRAG GTPases are required for lysosomal TSC2 recruitment (Demetriades et al., 2014; Menon et al., 2014; Carroll et al., 2016; Yang et al., 2020; Prentzell et al., 2021). 2) TSC1 binds lysosomal phosphatidylinositol-3,5-bisphosphate (PI3,5P2) via its N-terminal domain (Fitzian et al., 2021). The interplay of TSC2 and TSC1 tethering mechanisms in the lysosomal recruitment of the TSC complex remains to be determined. While the TSC complex is widely recognized as a transducer of insulin signals to RHEB and mTORC1 (Inoki et al., 2002; Hoxhaj and Manning, 2020), TSC complex association with the RRAG GTPases suggests also a responsiveness to amino acids (Demetriades et al., 2014; Menon et al., 2014; Carroll et al., 2016). Furthermore, several stressors including hypoxia, osmotic, pH and glycolytic stress enhance lysosomal recruitment of the TSC complex (Plescher et al., 2015; Demetriades et al., 2016). Hence, the control of the lysosomal localization of the TSC complex emerges as a central regulatory event that balances mTORC1 activity in response to growth factors, amino acids, and stresses.

### mTORC1 and Age-Related Stressors

Next to growth factors and amino acids, mammalian mTORC1 responds to a variety of stressors, including oxidative, DNA and unfolded protein stress (Heberle et al., 2015; Su and Dai, 2017; Ma et al., 2018). These stressors are connected to hallmarks of aging: 1) oxidative stress, promoted by the accumulation of reactive oxygen species (ROS), arises from dysfunctional oxidative phosphorylation in mitochondria (Desler et al., 2011), oxidative protein folding in the endoplasmic reticulum (ER) (Marciniak et al., 2004; Margittai and Sitia, 2011; Yoboue et al., 2018), and peroxisome metabolism (Titorenko and Terlecky, 2011). ROS accumulation results in oxidative damage of biomolecules including proteins and DNA (Liguori et al., 2018). 2) DNA stress is attributed to DNA damage, genome instability and telomere attrition (Maynard et al., 2015). DNA stress arises intrinsically from insufficient repair of replication errors or spontaneous hydrolytic reactions and telomere shortening during DNA replication (Maynard et al., 2015; Yousefzadeh et al., 2021), and upon damage by extrinsic agents, including electromagnetic radiation and chemical agents (Yousefzadeh et al., 2021). 3) ROS and DNA stress both promote proteasomal stress (loss of proteostasis) (Malhotra and Kaufman, 2007; Gonzalez-Quiroz et al., 2020), arising from imbalanced protein synthesis, folding, and turnover (declining autophagy and proteasome function) and resulting in an accumulation of unfolded proteins in the cytoplasm and/or in the ER (unfolded protein stress). The complex molecular mechanisms via which these stressors impinge on the mTORC1 network have been reviewed in detail by Heberle et al. (2015), Su and Dai (2017) and Ma et al. (2018).

Being mostly perceived as inhibitory, also activating stress inputs to the mTORC1 network have been reported that contribute to the delicate balance of mTORC1 activity under stress. In brief, oxidative stress inhibits mTORC1 by TSC complex-mediated RHEB-repression (Alexander et al., 2010; Zhang et al., 2013; Demetriades et al., 2016) and by inhibiting the lysosomal localization of mTORC1 (Yuan et al., 2015). ROS-mediated activation of mTORC1 also involves the TSC complex, as TSC1 and TSC2 are directly oxidized and inhibited by ROS (Yoshida et al., 2011). Furthermore, oxidative stress by mitochondrial ROS or arsenite activates mTORC1 via RAS (RAS proto-oncogene, GTPase) dependent activation of the PI3K-AKT1 pathway (Kim et al., 2018; Heberle et al., 2019). Arsenite also induces mTORC1 via the stress sensitive MAPK14 (mitogen-activated protein kinase 14, also known as p38) (Wang and Proud, 1997; Heberle et al., 2019) that directly phosphorylates the mTORC1 scaffold protein RPTOR (Wu et al., 2011) (Figure 1). UV-induced ROS activate mTORC1 via PI3K but independent of AKT1 via an unknown mechanism (Huang et al., 2002).

Unfolded protein stress inhibits mTORC1 via AKT1-repression (Qin et al., 2010; Kato et al., 2012; Li et al., 2018), for example through the negative AKT1 regulator TRIB3 (tribbles pseudokinase 3) (Ohoka et al., 2005). Prolonged unfolded protein stress triggered by the ER-stress inducers thapsigargin or tunicamycin inhibits AKT1 and phosphorylation of TSC2 at threonine 1462 and results in mTORC1 inactivation. In contrast, short-term unfolded protein stress mildly enhances AKT1 activity and phosphorylation of TSC2 (Di Nardo et al., 2009). Hence, activating and inhibitory cues converge on the TSC complex, depending on the stress duration and level. Interestingly, the RRAG GTPases may also contribute to unfolded protein stress sensing by mTORC1, as the ER stress inducer tunicamycin promotes RRAGC expression (Guha et al., 2017).

DNA stress is sensed via DNA damage response sensor proteins including PARP (poly ADP-ribose polymerase), ATM (ataxia telangiectasia mutated), DNA-PK (DNA protein kinase) and ATR (ataxia telangiectasia and Rad3 related) (Ma et al., 2018). PARP (Munoz-Gamez et al., 2009; Rodriguez-Vargas et al., 2012) and ATM (Alexander et al., 2010; Zhang et al., 2013; Ma et al., 2018) inhibit mTORC1 upon prolonged DNA stress by activating AMPK (AMP-activated protein kinase) which phosphorylates and activates TSC2, upstream of mTORC1 (Figure 1). Upon short term DNA stress (4 h etoposide), ATM/ATR activate mTORC1 by upregulating the level of MTOR, possibly by stabilizing the protein (Selvarajah et al., 2015). In contrast, prolonged DNA stress (24 h etopisode) results in mTORC1 inactivation and decreased MTOR protein levels (Selvarajah et al., 2015).

When active, mTORC1 enhances virtually all anabolic processes including protein synthesis, and inhibits catabolism, most notably autophagy [comprehensively reviewed by Ben-Sahra and Manning (2017), Rabanal-Ruiz and Korolchuk (2018), Tee (2018), Kim and Guan (2019), Liu and Sabatini (2020)]. Upon stress, mTORC1 suppression limits biosynthesis to essential processes needed for survival (Heberle et al., 2015), and enhances the degradation of cellular macromolecules and organelles by autophagy (Dossou and Basu, 2019), mitigating their damage and supplying the cell with intermediary metabolites as building blocks (Wong et al., 2020). Why does stress also elicit activating inputs to mTORC1? A certain level of tightly controlled mTORC1 activity may sustain processes required for stress survival (Thedieck et al., 2013; Heberle et al., 2015). This may concern the synthesis of stress response proteins (Chou et al., 2012; Hsieh et al., 2012; Thedieck et al., 2013) as well as the formation of SGs (Fournier et al., 2013; Mazan-Mamczarz et al., 2015; Sfakianos et al., 2018; Heberle et al., 2019), a stress-induced cytoplasmic compartment promoting survival (Reineke and Neilson, 2019). Hence, balanced mTORC1 activity might be required for SG-mediated cell survival and stress-recovery.

## SGs and mTORC1 Signaling

### Control of SG Formation by mTORC1

SGs are cytoplasmic non-membranous assemblies of proteins and mRNAs whose interaction involves liquid-liquid phase separation (LLPS) (Ivanov and Anderson, 2019). A rapidly growing field investigates the molecular mechanisms underlying SG formation (Van Treeck and Parker, 2018; Alberti et al., 2019; Mathieu et al., 2020; Peran and Mittag, 2020; Hofmann et al., 2021; Wiedner and Giudice, 2021). SG formation has been linked with different physiological consequences that are context-dependent and are currently under debate. Depending on the stress and its duration, SGs are rapidly turned over or they persist over long periods of time (Aulas et al., 2017; Markmiller et al., 2018). SGs buffer cellular stress by minimizing energy consumption [reviewed by Mahboubi and Stochaj (2017)] and by anti-apoptotic mechanisms (Arimoto et al., 2008; Takahashi et al., 2013; Thedieck et al., 2013). Such protective functions have been assigned to short-lived SGs (Reineke and Neilson, 2019). However, SGs might also exert pro-apoptotic effects (Fujimura et al., 2012; Aulas et al., 2018; Reineke and Neilson, 2019; Amen and Kaganovich, 2020) and they contribute to the formation of pathogenic protein aggregates (Aulas et al., 2018; Jeon and Lee, 2021). Chronic SG assembly has been linked with age-related disorders including neurotoxicity and cancer cell survival (Reineke and Neilson, 2019; Advani and Ivanov, 2020; Alberti and Hyman, 2021).

SGs form in a highly dynamic process within minutes upon stress exposure (Cao et al., 2020; Peran and Mittag, 2020). Via LLPS proteins and nucleic acids condense into liquid-like droplets surrounded by a liquid uncondensed environment (Alberti et al., 2019; Hofmann et al., 2021). LLPS involves the RNA content as well as proteins with LLPS-promoting domains such as RNA-binding domains (RBDs) (Alberti and Hyman, 2021; Hofmann et al., 2021) and intrinsically disordered regions (IDRs) (Alberti et al., 2019). The list of proteins that promote LLPS upon stress is rapidly growing (Youn et al., 2019). Early on, *bona fide* SG markers were defined based on the granular pattern that they acquire upon different stressors (Kedersha and Anderson, 2007). Some of these proteins were later shown to be required for SG assembly and are thus considered as core SG components (Kedersha et al., 2013). The core SG proteins include G3BP1/2 (Tourriere et al., 2003; Matsuki et al., 2013), TIA1 (TIA1 cytotoxic granule associated RNA binding protein) (Anderson and Kedersha, 2002; Gilks et al., 2004), and FMR1 (FMRP translational regulator 1) (Mazroui et al., 2002; Didiot et al., 2009). SG assembly is influenced by covalent modifications of RNAs and proteins that alter their physicochemical properties, such as surface charge, hydrophobicity, and binding strength between proteins and RNAs (Wiedner and Giudice, 2021). Post-translational modifications (PTMs) directly affecting SG formation include SUMOylation, methylation and phosphorylation (Kedersha et al., 2013; Mahboubi and Stochaj, 2017; Snead and Gladfelter, 2019; Cao et al., 2020). These PTMs are mediated by cellular signaling networks which thus directly impinge on SG assembly (Kedersha et al., 2013; Mahboubi and Stochaj, 2017; Reineke and Neilson, 2019).

**FIGURE 2:**
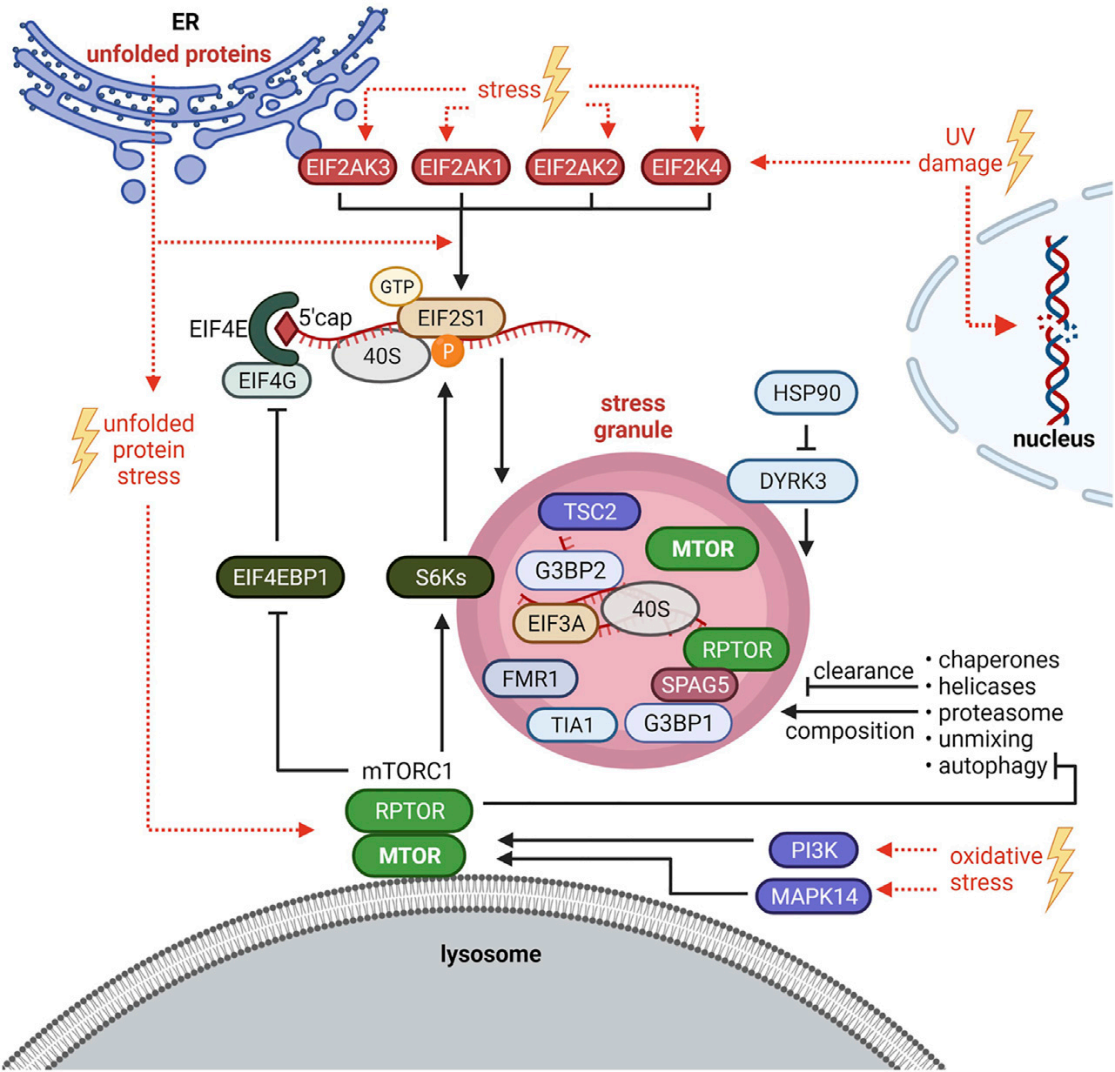
Crosstalk between stress granules (SGs) and mTORC1 signaling. SG formation is triggered by EIF2S1 phosphorylation at serine 51 or at the level of the EIF4F complex. HRI/EIF2AK1, PKR/EIF2AK2, PERK/EIF2AK3 and GCN2/EIF2AK4 phosphorylate EIF2S1-S51 in response to different stresses. Under oxidative stress, also the mTORC1-S6Ks axis enhances phosphorylation of EIF2S1-S51. Upon Bortezomib (unfolded protein stress), SGs are induced by mTORC1-driven phosphorylation of EIF4EBP1 to sustain EIF4F complex assembly. PI3K and MAPK14 activate mTORC1 to promote SG formation. Autophagy is inhibited by mTORC1 and mediates SG clearance and composition. SGs inhibit mTORC1 by sequestration of MTOR and RPTOR and via the HSP90-DYRK3 axis. SGs also recruit TSC2 and S6K1 and 2 (S6Ks). See main text for details and abbreviations.

SG formation is intimately linked with translation inhibition. When translation is inhibited, polysomes run off their mRNAs and “naked” mRNAs assemble with SG nucleating proteins to undergo LLPS (Kedersha et al., 2013; Hofmann et al., 2021). Under non-stress conditions, cap-dependent translation is initiated by the assembly of the EIF4F complex (EIF4E, EIF4G, EIF4B and EIF4A) at the 5′ 7-methylguanosine cap (5′ cap) of mRNAs (Sonenberg and Hinnebusch, 2009). One key regulatory event of EIF4F complex assembly is the phosphorylation of the eukaryotic translation initiation factor 4E-binding protein 1 (EIF4EBP1, also known as 4E-BP1) (Thoreen, 2017; Roux and Topisirovic, 2018; Tahmasebi et al., 2018). EIF4EBP1 competes with EIF4G for EIF4E binding, and prevents EIF4F complex assembly (Sonenberg and Hinnebusch, 2009). EIF4EBP1 phosphorylation by mTORC1 prevents EIF4EBP1-EIF4E binding and promotes EIF4F complex formation (Roux and Topisirovic, 2018) and recruitment of the 43S pre-initiation complex, consisting of the small ribosomal subunit (40S) bound to the eukaryotic translation initiation factor-2 complex (EIF2), GTP and Met-tRNA_i_^Met^ (Sonenberg and Hinnebusch, 2009). This complex is required for ribosome assembly and translation initiation (Merrick and Pavitt, 2018). EIF2 is a heterotrimeric complex consisting of alpha (EIF2S1), beta (EIF2S2), and gamma (EIF2S3) subunits (Merrick and Pavitt, 2018). EIF2S1 phosphorylation at serine 51 inhibits EIF2 (Sonenberg and Hinnebusch, 2009). Four kinases (HRI or EIF2AK1, PKR or EIF2AK2, PERK or EIF2AK3, GCN2 or EIF2AK4) phosphorylate EIF2S1-S51 in response to different stress situations (Donnelly et al., 2013) (Figure 2). This is considered as one of the main regulatory events for translation inhibition and SG initiation (Kedersha et al., 1999; Kedersha et al., 2002; Hofmann et al., 2021). However, SG formation can also be EIF2-independent, e.g. upon translation inhibition at the level of EIF4F complex assembly or activity (Hofmann et al., 2021).

mTORC1 inhibition has been proposed to initiate SG formation in mammalian cells (Fujimura et al., 2012; Hofmann et al., 2012; Panas et al., 2016) as it prevents the phosphorylation of EIF4EBP1 and thus the assembly of the EIF4F complex. In line with this idea, EIF4EBP1 shifts in size or is dephosphorylated, and increases its binding to the mRNA 5′ cap upon several stressors (H_2_O_2_, cold shock, selenite, nitric oxide) (Emara et al., 2012; Fujimura et al., 2012; Hofmann et al., 2012; Aulas et al., 2018). Based on observations that EIF4EBP1 or EIF4E inhibition by knockdown impairs SG formation upon selenite (Fujimura et al., 2012) or H_2_O_2_ (Emara et al., 2012) stress, respectively, EIF4EBP1-cap association has been proposed to enhance SG formation (Emara et al., 2012; Fujimura et al., 2012; Panas et al., 2016). Thus, unphosphorylated active EIF4EBP1 may promote SG assembly. However, none of the studies tested by inhibitors or knockdowns whether this process depends on mTORC1 (Emara et al., 2012; Fujimura et al., 2012; Hofmann et al., 2012). EIF4EBP1 is targeted by several kinases (Qin et al., 2016) and phosphatases (Kolupaeva, 2019) other than mTORC1. It remains thus open whether EIF4EBP1-mediated SG formation relates to inactive mTORC1, or if it is mediated by inactivation of another kinase or by a phosphatase. Sfakianos et al. (2018) showed that without stress, neither RPTOR knockdown nor rapamycin induced SGs. Hence, mTORC1 inhibition by itself is not sufficient to initiate SG formation. In contrast, mTORC1 inhibition has been shown by several studies to reduce SG formation upon heat shock, arsenite, and the proteasome inhibitor Bortezomib (Fournier et al., 2013; Mazan-Mamczarz et al., 2015; Sfakianos et al., 2018; Heberle et al., 2019). Conversely, TSC2 deficiency, known to hyperactivate mTORC1, increases the number of SGs formed upon arsenite or heat stress (Kosmas et al., 2021). As discussed above, mTORC1 activity under stress is enhanced–at least in part–by PI3Ks and MAPK14 (Heberle et al., 2019). Those kinases promote SG formation (Brown et al., 2011; Heberle et al., 2019), further supporting that a stress-activated signaling network converging on mTORC1 promotes SG formation.

All molecular mechanisms known so far to mediate mTORC1-driven SG formation impinge on the translation machinery. Upon arsenite, the kinases S6K1 and 2 (S6Ks) downstream of mTORC1 promote EIF2S1-S51 phosphorylation in mammalian cells (Sfakianos et al., 2018) (Figure 2), and this mechanism is conserved upon heat stress in the nematode *Caenorhabditis elegans*. Mammalian S6Ks enhanced SGs only under moderate arsenite stress. Higher concentrations abolished the S6Ks’ impact on SG formation, although it still depended on mTORC1 (Sfakianos et al., 2018). This might be explained by findings of Fournier et al. (2013) who showed that mTORC1-driven phosphorylation of EIF4EBP1 preserves EIF4E-EIF4G interaction, consequently enhancing SG formation upon high concentrations of arsenite as well as Bortezomib (Figure 2). Thus, mTORC1 enhances SG assembly via phosphorylation of S6Ks and EIF4EBPs, both events that are known to enhance translation (Roux and Topisirovic, 2018; Liu and Sabatini, 2020). This indicates that next to translation arrest (Van Treeck and Parker, 2018; Reineke and Neilson, 2019) activating signals to the translation machinery also contribute to SG formation.

Whether and which SGs form independently of mTORC1 remains to be investigated. To the best of our knowledge this has been so far claimed twice, for UV (Ying and Khaperskyy, 2020) and heat stress (Brown et al., 2011), based on the finding that MTOR inhibitors partially inhibit SG formation, but a certain fraction of cells with SGs remains. This observation is in agreement with several studies under different stresses (Fournier et al., 2013; Mazan-Mamczarz et al., 2015; Sfakianos et al., 2018; Heberle et al., 2019). The properties of SGs that are refractory to mTORC1 inhibition are therefore an intriguing topic for future studies. Where does mTORC1 control SG formation? mTORC1’s best described site of activity are the lysosomes (Rabanal-Ruiz and Korolchuk, 2018; Carroll, 2020). Recent evidence shows that SGs physically associate with lysosomes (Liao et al., 2019). Thus, lysosomal mTORC1 may enhance SG assembly. It is interesting to note that the core SG proteins G3BP1 and 2 have non-granule functions as mTORC1 suppressors at lysosomes (Prentzell et al., 2021). It remains open whether the G3BP pools at lysosomes and SGs are separate, or whether G3BPs shuttle between these two compartments. Lysosomes have also been linked to SGs in the context of autophagy: as autophagy degrades aggregated proteins (Yang and Klionsky, 2020) it is straightforward to assume that autophagy contributes to SG clearance. Indeed this has been reported in mammals (Ryu et al., 2014; Marrone et al., 2018; Silva et al., 2019), *Saccharomyces cerevisiae* (Buchan et al., 2013) and *Caenorhabditis elegans* (Zhang et al., 2018). mTORC1 is a key suppressor of autophagy (Rabanal-Ruiz et al., 2017). Thus, mTORC1 may enhance SG assembly, at least in part, by inhibiting autophagy. Furthermore, autophagy not only controls SG turnover (Buchan et al., 2013; Lee, 2015) but also their composition (Seguin et al., 2014; Advani and Ivanov, 2020) (Figure 2). This raises the possibility that mTORC1 affects SG composition by inhibiting autophagy.

On a broader level, SG clearance and composition is affected not only by autophagy but also by chaperones, RNA helicases, the proteasomal machinery and unmixing of LLPS condensates (Alberti et al., 2017; Alberti and Hyman, 2021). The cooperation of these processes in SG dynamics is currently investigated by a highly active and growing field of research. The interplay of mTOR with the proteasome is also a matter of active scientific debate (Adegoke et al., 2019). It will be intriguing to link these fields and unravel mTOR’s role in SG turnover.

### SGs Inhibit mTORC1

Not only does mTORC1 regulate SG formation and clearance. Conversely, SGs also inhibit mTORC1 by several mechanisms in yeast as well as in mammalian cells (Takahara and Maeda, 2012; Thedieck et al., 2013; Wippich et al., 2013; Mediani et al., 2021). In mammalian cells, SG recruitment of RPTOR is mediated by SPAG5 (sperm associated antigen 5, also known as astrin) and leads to the disassembly and inhibition of mTORC1 (Thedieck et al., 2013) (Figure 2). MTOR localizes to SGs too (Wippich et al., 2013), but the molecule mediating this recruitment is unknown (Figure 2). Likewise, the *Saccharomyces cerevisiae* RPTOR orthologue KOG1 and TOR1 localize to SGs (Takahara and Maeda, 2012). Also the TSC subunit TSC2 (Kosmas et al., 2021) and the mTORC1 substrates S6K1 and 2 (Sfakianos et al., 2018) have been recently reported at SGs (Figure 2), which might impinge on mTORC1 activity as well. It will be interesting to delineate the coordination of the recruitment and disassembly of the TSC and mTORC1 complexes and their substrates at SGs. SGs also regulate mTORC1 via the kinase DYRK3 (dual specificity tyrosine phosphorylation regulated kinase 3) (Wippich et al., 2013; Mediani et al., 2021). DYRK3 binds HSP90 (heat shock protein 90 family) an essential chaperone which regulates the folding and stability of many clients including stress response factors important to resolve a variety of proteotoxic stresses [reviewed in detail by Schopf et al. (2017), Calderwood (2018), Moran Luengo et al. (2019), Lang et al. (2021)]. Under non-stress conditions, HSP90 keeps DYRK3 in an active confirmation. DYRK3 phosphorylates the mTORC1 inhibitor AKT1S1 at threonine 246, thus de-repressing mTORC1 (Wippich et al., 2013). Upon stress or HSP90 inhibition (Wippich et al., 2013; Mediani et al., 2021), inactive DYRK3 is recruited to SGs via its N-terminal IDR, resulting in AKT1S1 activation and mTORC1 inhibition. SG-localized DYRK3 also stabilizes SGs, thereby enhancing their inhibitory effect on mTORC1. As mTORC1 is inhibited by SGs, mTORC1-driven SG formation may constitute a negative feedback mechanism that restricts mTORC1 activation by stress, contributing to the fine-tuning of cellular anabolism and catabolism that maintains cellular homeostasis under stress.

## Discussion

### Linkage of MTOR and SGs in Aging

Stresses linked to hallmarks of aging (see *mTORC1 and Age-Related Stressors*) impinge on mTORC1 activity and SG formation (see *SGs and mTORC1 Signaling*). Conversely, enhanced mTORC1 signaling (Papadopoli et al., 2019) and SG formation (Cao et al., 2020) have been linked to age-related processes, and MTOR and SG levels often correlate with the severity of age-related diseases (Papadopoli et al., 2019; Liu and Sabatini, 2020; Chrienova et al., 2021). However, the crosstalk between mTORC1 and SG formation in the context of aging progression is poorly explored.

Aging is characterized by increased numbers of senescent cells that have been assigned to stress stimuli which result in irreversible cell cycle arrest (Lopez-Otin et al., 2013; Papadopoli et al., 2019). Senescent cells are impaired in their proliferative capacity, but they maintain an active metabolism (Papadopoli et al., 2019) and exhibit reduced apoptosis (Wanner et al., 2020) despite stress-induced damage (Song et al., 2020). Although SG formation is mainly recognized to counteract senescence (Omer et al., 2018; Cao et al., 2020), senescent cells can form SGs (Lian and Gallouzi, 2009). Interestingly, senescent cells exhibit mTORC1 uncoupling from its suppressors, resulting in mTORC1 hyperactivity (Carroll et al., 2017). Hence, chronic mTORC1 activity might sensitize senescent cells to SG formation. In line with this, senescent cells present a higher number of SGs upon acute stress (Lian and Gallouzi, 2009) and show slowed SG disassembly after stress recovery (Gallouzi, 2009; Lian and Gallouzi, 2009). SGs enhance survival by sequestering pro-apoptotic proteins (Arimoto et al., 2008). mTORC1-driven SG formation may also exert a negative feedback on mTORC1 that restricts its excessive activity, known to result in apoptosis (Appenzeller-Herzog and Hall, 2012; Thedieck et al., 2013). Hence, SG formation might contribute to the survival and increased presence of senescent cells in aging tissues by sequestering pro-apoptotic factors and by dampening mTORC1 activity.

Neurodegeneration and cancer are age-related diseases associated with senescence (Baker and Petersen, 2018; Sikora et al., 2021). Both aberrant mTORC1 activity (Chrienova et al., 2021; Querfurth and Lee, 2021) and chronic SG formation (Wolozin and Ivanov, 2019; Asadi et al., 2021) link with neurodegenerative diseases including ALS (amyotrophic lateral sclerosis), FTD (frontotemporal dementia), AD (Alzheimer’s disease) or PD (Parkinson’s disease). However, their crosstalk in neurodegenerative diseases is largely unknown. It is conceivable that hyperactive mTORC1 drives chronic SG formation, and thereby promotes the progression of neurodegeneration. In cancer, MTOR is widely recognized as a key driver and drug target (Mossmann et al., 2018; Liu and Sabatini, 2020), whereas the importance of SG proteins for tumorigenesis and treatment response is only beginning to emerge (Anderson et al., 2015; Gao et al., 2019). Many SG proteins are dysregulated in cancer (Adjibade et al., 2015; Somasekharan et al., 2015; Vilas-Boas Fde et al., 2016; Sim et al., 2019) and their altered expression has been linked with drug response and disease outcome (Gao et al., 2019). The role of mTORC1-SG crosstalk in neurodegeneration and cancer therefore deserves in depth investigation regarding its role in therapy response and to develop new therapy concepts.

## Conclusion

The mTORC1 cascade and SGs are key mediators of cell growth and survival that are closely intertwined in a network whose complexity we are only beginning to understand. Current research on MTOR signaling and on SGs is largely confined to separate fields. Comparatively few studies tackle their interplay and are often correlative in nature. Toward a comprehensive understanding, the challenge of investigating mechanistic inhibitory and activating links between the SG and MTOR networks will be key to identify causal relationships between them. These mechanisms may provide leads for treatments that account for specific metabolic alterations and stresses in age-related conditions such as cellular senescence, cancer, and neurodegeneration.
